# Sustained decline in birth weight and increased rate of preterm infants born small for gestational age in Japan

**DOI:** 10.3389/fped.2024.1480527

**Published:** 2024-09-30

**Authors:** Akinori Moriichi, Erika Kuwahara, Narumi Kato

**Affiliations:** Division of Information for Specific Pediatric Chronic Diseases, Research Institute, National Center for Child Health and Development, Tokyo, Japan

**Keywords:** preterm birth, premature, low birth weight, nulliparous, parity, advanced maternal age

## Abstract

**Background:**

Birth weights have continued to decline in Japan in recent years. However, secular trend changes such as the birth weight relative to the week of gestation remain to be explored. This study aimed to determine the trends over time in mean birth weight and small for gestational age (SGA) rate for each gestational week.

**Methods:**

We used a large dataset of 27,015,792 births obtained from birth certificates between 1997 and 2021. Births from 22 to 41 weeks of gestation were evaluated in six groups (22–24, 25–27, 28–31, 32–33, 34–36, and 37–41 weeks of gestational age). For each group, secular trend changes in the *z*-scores calculated from standard birth weight values were assessed. Time trends in the proportion of SGA and mean birth weight *z*-scores were evaluated using the Cochran–Armitage trend test and linear regression analysis. Binomial logistic regression was performed to ascertain the effects of gestational age, sex, primiparity, number of births, and maternal age on the likelihood of SGA.

**Results:**

The mean birth weight of preterm infants continued to decrease, and the *z*-score for mean birth weight decreased linearly, falling to −0.7 at 25–27 weeks of gestation from 1997–2001 (first period) to 2017–2021 (final period). Maternal age continued to increase from the first period to the last period for all weeks of gestation. There was a linear increase in the SGA rate in preterm infants born at <34 weeks. Odds ratios for the likelihood of SGA were 1.3 times higher for maternal age ≥40 years than that for 25–29 years (95% CI: 1.29–1.33, *p* < 0.001).

**Conclusions:**

In Japan, there has been a continuous decline in birth weight and an increase in the rate of preterm SGA infants.

## 1 Introduction

Small-for-gestational-age (SGA) infants have a higher mortality risk than that of infants born at appropriate gestational age due to heart, respiratory, and digestive diseases, as well as accidents, suicide, and homicide in all age groups through adulthood (1). Moreover, SGA infants have higher rates of bronchopulmonary dysplasia, cerebral palsy, developmental delay, and visual impairment compared to infants born at appropriate gestational age (2, 3), making an increased SGA rate a significant public health issue.

While increased birth weight has been reported in England and Wales (4), the converse has been observed in France, Germany, and the United States since the 2000s (5–7). Notably, in Japan, the average birth weight has declined, partly due to a shift in the peak birth period from 38 to 37 weeks (8). Although the proportion of preterm infants has not changed significantly in recent years (8, 9), the birth weight of preterm infants may have decreased over time (10). Nevertheless, there have been few investigations on the changes in the rate of preterm births and trends in average birth weight by week of gestation.

The maternal age at birth has increased in many high-income countries. The mean maternal age at birth exceeded 30 years in Spain and Italy in the late 1990s; Denmark, Sweden, and Switzerland in the early 2000s; and Finland, Germany, South Korea, Taiwan, and Japan in the late 2000s and has continued to increase in several other countries (11). Advanced maternal age leads to increased rates of preterm birth, low birth weight (LBW), and SGA (12–19). At advanced maternal age, the quality of oocytes is likely to have declined (20), and placental function may be impaired (21), which may be associated with preterm delivery, fetal growth restriction, and the rate of SGA.

This study aimed to clarify the secular trends in birth weight and SGA rates by weeks of gestation between 1997 and 2021 in Japan and to assess the impact of advanced maternal age.

## 2 Materials and methods

### 2.1 Data source

This study was based on Japanese birth certificates, which contain data on all women who gave birth in Japan. Birth certificate data from January 1, 1997, to December 31, 2021, were obtained from the Ministry of Health, Labour and Welfare. Birth certificates provide exhaustive national data collected from all births in Japan under the Family Register Act. Data were anonymous and included the infant's sex, year and place of birth, birth weight and length, singleton or multiple births, weeks of gestation at birth, maternal parity, and nationality. The method of delivery was not included. For comparison with standard somatic measures, the population analyzed in this study was restricted to infants of Japanese nationality. The study protocol was approved by the Ethics Committee of the National Center for Child Health and Development, Tokyo, Japan (No. 2023–230).

### 2.2 Estimation of weeks of gestation

All obstetric facilities in Japan have had ultrasound systems since the 1980s (22). Nearly all pregnancies were diagnosed and managed using ultrasonography throughout the study period. The results of the last menstrual period and ultrasound measurements were used to determine the number of pregnancy weeks.

### 2.3 Data analyses

Data were divided into five periods (1997–2001, 2002–2006, 2007–2011, 2012–2016, and 2017–2021). We excluded cases with unknown birth year, gestational week, birth weight, and maternal age from the database of birth certificates. *Z*-scores of birth weights for cases between 22 and 41 weeks of gestation were calculated. The primary outcome was the *z*-score of the mean birth weight, calculated using Japanese sex-specific standard birth weights by gestational age. The percentage of SGA was defined as less than −2 SD of birth weight and was computed using growth curves derived from standard birth weight (23). The *z*-score of the mean birth weight was evaluated for six gestational week groups: group 1, 22–24 gestational weeks; group 2, 25–27 gestational weeks; group 3, 28–31 gestational weeks; group 4, 32–33 gestational weeks; group 5, 34–36 gestational weeks; and group 6, 37–41 gestational weeks. In addition, the relationship between maternal and perinatal factors and the *z*-score of the mean birth weight was analyzed. Cases with *z*-scores ≤−5 SD or ≥+5 SD were excluded due to the possibility of incorrectly recording outliers. Obstetric practice guidelines in Japan recommend avoiding births past term at 42 weeks or more of gestation. Consequently, no normative birth weight value is available at 42 weeks or more. Therefore, past-term births were excluded from the study.

### 2.4 Statistical analysis

Data are presented as mean (standard deviation) or number of cases (percentage). Cochran–Armitage or linear regression was used to analyze trends across the five time periods for maternal age, percentage of primipara, multiple births, preterm births, and SGA infants. Binomial logistic regression analysis was performed to estimate the relative contribution of maternal and perinatal factors to the likelihood of an SGA child: gestational week, gender, maternal age, number of deliveries, multiple births, and maternal age as independent variables. We also included them as independent variables in multivariate models. Adjusted odds ratios and 95% confidence intervals (CIs) were used to evaluate the association between SGA and clinical features. All tests were two-tailed, and *P* < 0.05 was used to define statistical significance. Statistical analyses were performed using the SPSS software version 27.1 (IBM Corp., Armonk, NY, USA).

## 3 Results

A total of 27,015,792 births were registered on birth certificates from January 1, 1997, to December 31, 2011. Of the 27,015,792 cases, 840,759 were excluded as 349,219 were non-Japanese, 346,358 had an undetermined gestational week, 131,954 were born at gestational weeks <22 weeks or >41 weeks, the birth weight of 5,977 was unknown, 70 had no maternal age data, and 7,181 had a birth weight *z*-score <−5SD or >+5SD. Therefore, 26,175,033 cases were included in this study.

Table 1 shows the characteristics of births in the first (1997–2001) and last (2017–2021) periods examined. The average number of live births per year gradually decreased throughout the study period from 1,174,067 in the first period to 876,323 in the last period. The maternal age was persistently elevated at all weeks of gestation, according to the more recent data. The percentage of full-term births at 37 weeks or more of gestation decreased slightly from 94.8% in 1997 to 94.4% in 2021, with a slight increase in preterm births. The most significant percentage increase was observed in late preterm births at 34–36 weeks, from 4.1% in 1997 to 4.4% in 2021, with only a slight increase in the other gestational weeks. The number of primiparae below 32 gestational weeks gradually increased over the years (*P* < 0.001), with a decrease after 32 weeks of gestation (*P* < 0.001). There was a slight decrease (*P* < 0.001) in the proportion of multiple births at all weeks of gestation over time. A linear decrease in the mean birth weight was observed at all weeks of gestation in both sexes. Preterm births at <37 gestational weeks have shown a continued decline in mean birth weight *z*-scores over the past 25 years.

**Table 1:** Comparison of perinatal variables between the first period (1997–2001) and final period (2017–2021).

Variables			Gestational age	1997–2001 (*n* = 5,870,337)		2017–2021 (*n* = 4,381,617)		*P*-value
Maternal age	All births	years, mean (SD)	22–24 weeks	29.6	(5.2)	32.3	(5.5)	<0.001
			25–27 weeks	29.6	(5.2)	32.5	(5.5)	<0.001
			28–31 weeks	29.7	(5.1)	32.6	(5.4)	<0.001
			32–33 weeks	29.6	(5.0)	32.5	(5.4)	<0.001
			34–36 weeks	29.5	(4.8)	32.2	(5.3)	<0.001
			37–41 weeks	28.9	(4.5)	31.5	(5.1)	<0.001
Primipara	All births	*n*, %	22–24 weeks	1,370	40.0%	1,817	46.6%	<0.001
			25–27 weeks	3,591	40.2%	3,252	46.3%	<0.001
			28–31 weeks	10,390	41.6%	8,336	42.9%	<0.001
			32–33 weeks	12,754	42.7%	10,053	41.8%	0.419
			34–36 weeks	104,842	44.0%	80,779	42.2%	<0.001
			37–41 weeks	2,706,420	48.6%	1,921,571	46.5%	<0.001
Multiple births	All births	*n*, %	22–24 weeks	629	18.4%	593	15.2%	<0.001
			25–27 weeks	1,877	21.0%	1,207	17.2%	<0.001
			28–31 weeks	5,687	22.7%	4,379	22.5%	0.936
			32–33 weeks	6,913	23.2%	5,725	23.8%	0.001
			34–36 weeks	40,492	17.0%	33,816	17.6%	<0.001
			37–41 weeks	57,273	1.0%	44,077	1.1%	<0.001
Birth weight	Boys	g, mean (SD)	22–24 weeks	633	(116)	591	(111)	<0.001
			25–27 weeks	903	(186)	860	(195)	<0.001
			28–31 weeks	1,413	(365)	1,351	(312)	<0.001
			32–33 weeks	1,883	(357)	1,838	(326)	<0.001
			34–36 weeks	2,480	(395)	2,445	(372)	<0.001
			37–41 weeks	3,127	(380)	3,101	(370)	<0.001
	Girls	g, mean (SD)	22–24 weeks	593	(109)	548	(111)	<0.001
			25–27 weeks	841	(175)	798	(183)	<0.001
			28–31 weeks	1,349	(390)	1,275	(302)	<0.001
			32–33 weeks	1,789	(367)	1,737	(325)	<0.001
			34–36 weeks	2,384	(399)	2,348	(375)	<0.001
			37–41 weeks	3,036	(371)	3,005	(361)	<0.001
SGA	Boys	*n*, %	22–24 weeks	62	3.3%	175	8.5%	<0.001
			25–27 weeks	4,257	3.3%	3,118	8.5%	<0.001
			28–31 weeks	12,030	12.1%	9,181	14.7%	<0.001
			32–33 weeks	15,472	8.8%	12,232	9.4%	<0.001
			34–36 weeks	129,391	4.6%	104,053	4.4%	<0.001
			37–41 weeks	2,790,366	1.9%	2,076,527	1.4%	<0.001
	Girls	*n*, %	22–24 weeks	58	3.7%	164	8.9%	<0.001
			25–27 weeks	435	10.3%	611	18.4%	<0.001
			28–31 weeks	1,508	13.3%	1,287	14.9%	<0.001
			32–33 weeks	1,319	10.3%	1,173	11.1%	<0.001
			34–36 weeks	5,542	5.4%	4,260	5.1%	<0.001
			37–41 weeks	49,927	1.8%	28,399	1.4%	<0.001

SGA, small for gestational age, defined as birth weight <−2 SD. Continuous data were assessed using the *t*-test. The maternal age was higher in the final period (2015–2021) than in the first period (1995–1999) for all weeks of gestation (*P* < 0.001). Birth weights for both sexes were lower in the final period than in the first period for all weeks of gestation. Categorical data were assessed using the chi-square test. The percentage of primipara and multiple births increased linearly in preterm births less than 32 weeks of gestation. A linear increase in the rate of SGA was observed in preterm infants (<37 weeks) for both sexes.

**Figure 1:**
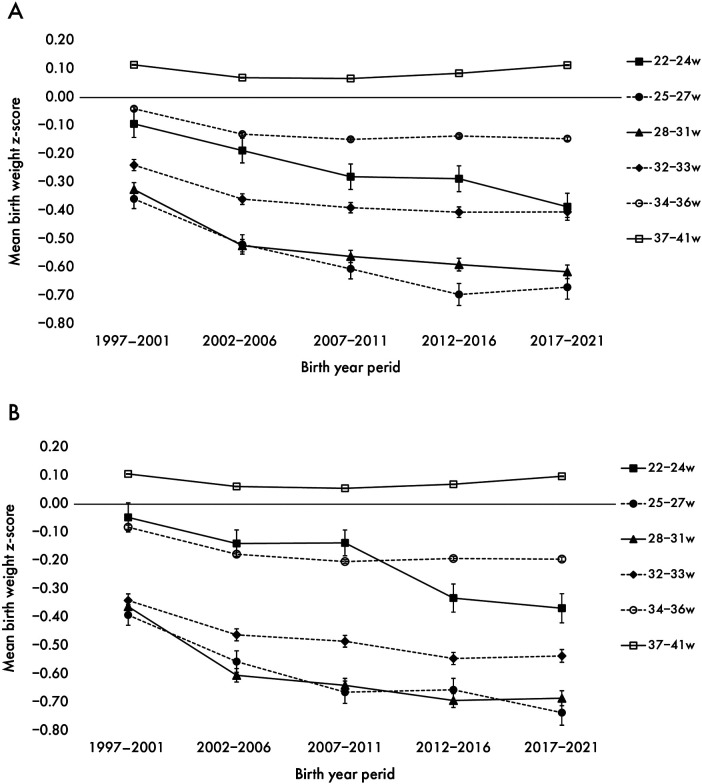
Secular trend in *z*-score of mean birth weight by weeks of gestation for boys **(A)** and girls **(B)** the trends in mean *z*-score of birth weight from the first period (1997–2001) to the last period (2017–2021) with 95% confidence intervals by sex.

From the first period (1997–2001) to the final period (2017–2021), the mean birth weight *z*-scores decreased by −0.31 at 22–24 weeks, −0.70 at 25–27 weeks, −0.65 at 28–31 weeks, −0.46 at 32–33 weeks, and −0.11 at 34–36 weeks' gestation. At 34–36 weeks of gestation, the *z*-score decreased until 2007 and then plateaued. For full-term births (37–41 weeks), the mean birth weight *z*-score decreased until 2007, with subsequent recovery later (Figure 1).

**Figure 2:**
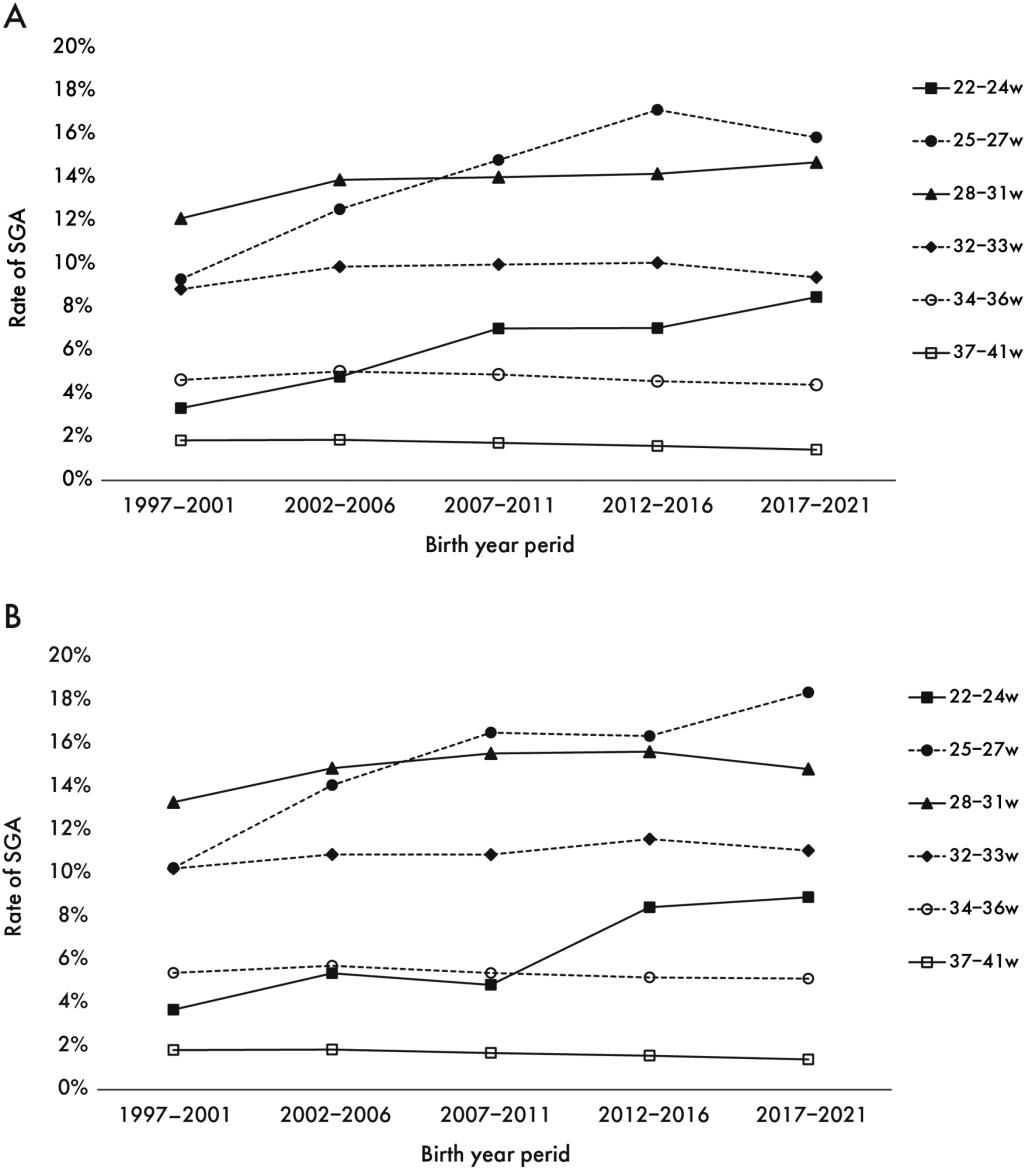
Secular trend in the SGA rate for boys **(A)** and girls **(B)** the trends in the percentage of SGA from the first period (1997–2001) to the last period (2017–2021) by sex.

There was a marked increase in the SGA rate over time in both sexes at less than 32 weeks of gestation. In contrast, the SGA rate in late preterm (34–36 weeks) and full-term births decreased since 2007. Throughout all birth years, the SGA rate tended to be higher in girls than in boys among preterm infants born at ≥25 weeks' gestation (Figure 2).

**Figure 3:**
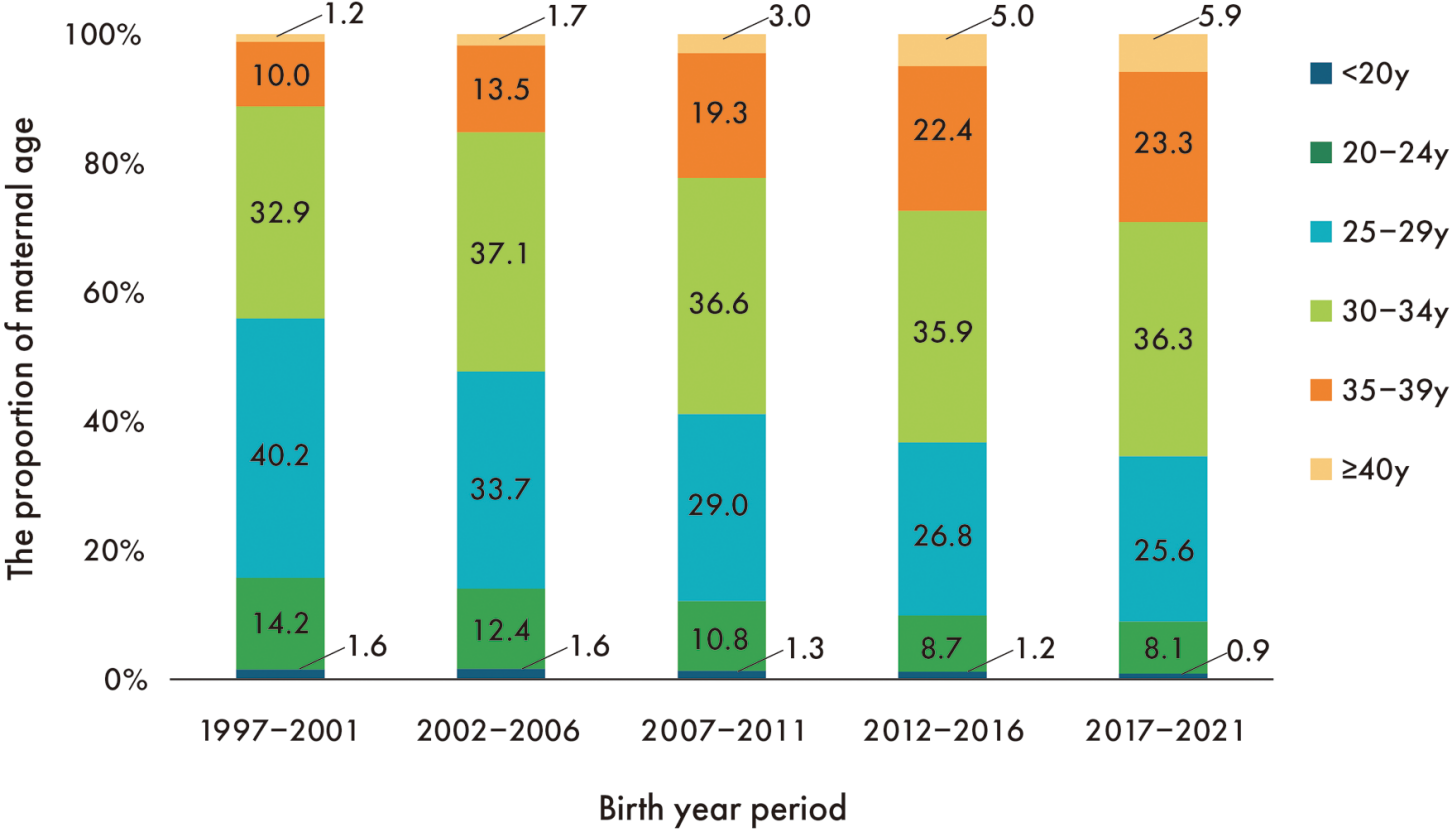
Secular trend in the proportion of maternal age. The proportion of mothers <30 years decreased linearly throughout the entire period, while the proportion of mothers ≥30 years increased linearly.

Maternal age increased secularly in all gestational age groups, with preterm infants having a higher maternal age than full-term infants. The percentage of maternal ages 35–39 increased 2.3-fold from the first to the last period, while the percentage of maternal ages ≥40 increased 4.9-fold (Figure 3).

Binomial logistic regression was performed to ascertain the effects of gestational age, sex, primiparity, multiple births, and maternal age on the likelihood of SGA. The five predictive variables were statistically significant (Table 2). Preterm births and first births were more likely to be SGA. Multiple births had 4.3 times higher odds of being SGA (95% CI: 4.25–4.34, *P* < 0.001). Increased maternal age was associated with an increased likelihood of SGA. The odds ratio for SGA was 1.3 times higher for maternal age ≥40 years than for age 25–29 years, demonstrating an increase with advancing maternal age (95% CI: 1.29–1.33, *P* < 0.001).

**Table 2:** Logistic regression predicting the likelihood of SGA.

Variables		Univariate			Multivariate		
		Odds ratio (95% CI)		*P*-value	Odds ratio (95% CI)		*P*-value
Gestational week		0.83	(0.83–0.83)	<0.001	0.87	(0.87–0.87)	<0.001
Male		0.99	(0.98–0.99)	<0.001	1.00	(0.99–1.00)	0.079
Primipara		1.02	(1.02–1.03)	<0.001	1.12	(1.11–1.13)	<0.001
Multiple births		6.72	(6.66–6.78)	<0.001	4.30	(4.25–4.34)	<0.001
Maternal age, years	<20	0.96	(0.94–0.99)	0.005	0.95	(0.92–0.97)	<0.001
	20–24	1.00	(0.99–1.01)	0.452	1.01	(1.00–1.02)	0.006
	25–29	1 [Reference]			1 [Reference]		
	30–34	1.05	(1.04–1.06)	<0.001	1.03	(1.02–1.03)	<0.001
	35–39	1.19	(1.18–1.19)	<0.001	1.12	(1.11–1.13)	<0.001
	≥40	1.43	(1.41–1.45)	<0.001	1.31	(1.29–1.33)	<0.001

SGA, small for gestational age, defined as birth weight <−2 SD. The odds ratio was adjusted for perinatal confounders (gestational age, sex, primipara, multiple births, maternal age, and birth year period). The odds ratios for the likelihood of SGA were higher for primipara and multiple births. Compared to the group of maternal age of 25–29 years, the group over 40 years old had a higher odds ratio for the likelihood of SGA.

## 4 Discussion

This study investigated secular trends in birth weight by week of gestation and explored factors affecting birth weight using birth certificate data from the past 25 years in Japan. The results showed that the mean birth weight of preterm infants has declined, and the *z*-score for mean birth weight decreased linearly from 1997 to 2001 (first period) to 2017–2021 (last period), whereas that of full-term infants gradually recovered from around 2010 onwards. Moreover, there was a linear increase in the SGA rate in preterm infants born at <34 weeks. A mild increase in the preterm birth rate was observed. Odds ratios for the likelihood of SGA were 1.3 times higher for maternal age ≥40 years than that for 25–29 years. Our data suggests that advanced maternal age and increased primiparous birth rates in preterm infants may be associated with increased SGA rates.

To our knowledge, no study has reported on a longitudinal decrease in birth weight in preterm infants. For a comparative analysis, we subdivided all Japanese births into gestational age categories—22–24, 25–27, 28–31, 32–33, 34–36, and 37–41 weeks. The causes of SGA, LBW, and preterm birth include maternal, fetal, and environmental factors, such as living environment, malnutrition, healthcare systems, and education (24). However, it is unlikely that environmental factors in Japan changed markedly during the study period, implying that maternal and fetal factors are likely to be more influential. These factors include primiparity, multiple births, and young or advanced maternal age (25–28). Our study did not demonstrate a notable increase in the rate of primiparas or multiple births during the study period. The percentage of mothers aged <20 years reduced by 50% between 1995 and 2021. Maternal age increased linearly throughout the study period for all gestational weeks. A large United States of America population study indicated that women aged ≥40 had significantly higher SGA rates than those aged 20–29 (16). Likewise, a Swedish study using birth registration data showed that maternal age ≥35 years had a higher risk of increased rates of SGA compared to maternal age of 20–29 years (17). Previous studies have indicated that primiparous women have higher SGA (16, 18, 26, 27). In the present study, the odds ratio for SGA was higher for maternal age ≥40 years than for age 25–29 years, and primipara was also associated with a higher odds ratio for SGA. Furthermore, a dose-dependent relationship was found between advanced maternal age and the odds ratio for SGA infants. A large population-based study using data from Denmark, Sweden, Norway, and Finland found an increased risk of LBW and preterm birth with increasing maternal age in all four countries (12). These data are supported by a systematic review showing that maternal age ≥35 years is associated with a higher risk of increased rates of LBW and preterm birth (14).

We revealed conflicting findings; the birth weight of preterm infants continued to decline, whereas the number of full-term infants recovered in recent years. The guidelines of the Japan Society of Obstetrics and Gynecology in the 1980s and 1990s identified the prevention of preeclampsia as a top priority, and rigorous weight gain control was implemented in pregnant women (29). Due to the suspicion that the year-to-year decline in birth weight may be related to maternal weight gain restrictions, the Ministry of Health and Welfare revised the guidelines in 2006 to relax weight control (29). The mean birth weight increased slightly from 2010 onwards at 37–41 weeks of gestation, which may have been influenced by the revised guidelines. However, the reason for the continued decline in the mean birth weight of preterm infants, especially for those born at less than 34 weeks, remains unclear.

This study has some limitations. The data on birth certificates did not include the mode of delivery, smoking, parental height, socioeconomic status, and educational attainment of the parents. Furthermore, information on maternal body mass index and weight gain during pregnancy, which are essential determinants of SGA, was unavailable. In addition, birth certificates do not contain medical information, including complications or malformations in neonates and maternal illness or complications. Therefore, we could not differentiate cases based on the medical status of the infants. Nevertheless, the selection bias in this study was expected to be sufficiently small due to the extensive data analysis using birth certificates for all births in Japan.

In conclusion, in Japan, the birth weight of preterm infants continues to decrease while the rate of SGA infants is increasing. Obstetricians and neonatologists should be aware of this fact, and further research on the risk factors for SGA infants is warranted.

## Data Availability

The datasets presented in this article are not readily available because they were provided by the Ministry of Health, Labour and Welfare (MHLW) under usage restrictions. Requests to access the datasets should be directed to the MHLW, Japan (www-admin@mhlw.go.jp).

## References

[1] Wennerström ECM Simonsen J Melbye M. Long-term survival of individuals born small and large for gestational age. PLoS One. (2015) 10:e0138594. 10.1371/journal.pone.0138594 26390219 PMC4577072

[2] Suenaga H Nakanishi H Uchiyama A Kusuda S, Neonatal research network of Japan. Small for gestational age affects outcomes on singletons and inborn births in extremely preterm infants: a Japanese cohort study. Am J Perinatol. (2022) 41:e780–7. 10.1055/a-1933-4627 36041470

[3] Castanys-Muñoz E Kennedy K Castañeda-Gutiérrez E Forsyth S Godfrey KM Koletzko B Systematic review indicates postnatal growth in term infants born small-for-gestational-age being associated with later neurocognitive and metabolic outcomes. Acta Paediatr. (2017) 106:1230–8. 10.1111/apa.13868 28382722 PMC5507303

[4] Ghosh RE Berild JD Sterrantino AF Toledano MB Hansell AL. Birth weight trends in England and Wales (1986–2012): babies are getting heavier. Arch Dis Child Fetal Neonatal Ed. (2018) 103:F264–70. 10.1136/archdischild-2016-311790 28780501 PMC5916100

[5] Diouf I Charles MA Blondel B Heude B Kaminski M. Discordant time trends in maternal body size and offspring birthweight of term deliveries in France between 1972 and 2003: data from the French national perinatal surveys. Paediatr Perinat Epidemiol. (2011) 25:210–7. 10.1111/j.1365-3016.2010.01188.x 21470260

[6] Donahue SMA Kleinman KP Gillman MW Oken E. Trends in birth weight and gestational length among singleton term births in the United States: 1990–2005. Obstet Gynecol. (2010) 115:357–64. 10.1097/AOG.0b013e3181cbd5f5 20093911 PMC3219436

[7] Schiessl B Beyerlein A Lack N Von Kries R. Temporal trends in pregnancy weight gain and birth weight in Bavaria 2000–2007: slightly decreasing birth weight with increasing weight gain in pregnancy. J Perinat Med. (2009) 37:374–9. 10.1515/JPM.2009.068 19309253

[8] Yoshida H Kato N Yokoyama T. Early full-term birth is an important factor for the increase in the proportion of low-birthweight infants between 1980 and 2015 in Japan. J Natl Inst Public Health. (2022) 71:77–86. 10.20683/jniph.71.1_77

[9] Sakata S Konishi S Ng CFS Watanabe C. Preterm birth rates in Japan from 1979 to 2014: analysis of national vital statistics. J Obstet Gynaecol Res. (2018) 44:390–6. 10.1111/jog.13460 28901036

[10] Kato N Sauvaget C Yoshida H Yokoyama T Yoshiike N. Factors associated with birthweight decline in Japan (1980–2004). BMC Pregnancy Childbirth. (2021) 21:337. 10.1186/s12884-021-03819-0 33906616 PMC8080357

[11] OECD. OECD family database. Available online at: https://www.oecd.org/els/family/database.htm (Accessed July 26, 2024)

[12] Aradhya S Tegunimataka A Kravdal Ø Martikainen P Myrskylä M Barclay K Maternal age and the risk of low birthweight and pre-term delivery: a pan-nordic comparison. Int J Epidemiol. (2023) 52:156–64. 10.1093/ije/dyac211 36350574 PMC9908063

[13] Valero De Bernabé J Soriano T Albaladejo R Juarranz M Calle ME Martínez D Risk factors for low birth weight: a review. Eur J Obstet Gynecol Reprod Biol. (2004) 116:3–15. 10.1016/j.ejogrb.2004.03.007 15294360

[14] Lean SC Derricott H Jones RL Heazell AEP. Advanced maternal age and adverse pregnancy outcomes: a systematic review and meta-analysis. PLoS One. (2017) 12:e0186287. 10.1371/journal.pone.0186287 29040334 PMC5645107

[15] Auger N Hansen AV Mortensen L. Contribution of maternal age to preterm birth rates in Denmark and Quebec, 1981–2008. Am J Public Health. (2013) 103:e33–8. 10.2105/AJPH.2013.301523 23947312 PMC3780760

[16] Palatnik A De Cicco S Zhang L Simpson P Hibbard J Egede LE. The association between advanced maternal age and diagnosis of small for gestational age. Am J Perinatol. (2020) 37:37–43. 10.1055/s-0039-1694775 31430823 PMC8104456

[17] Cnattingius S Forman MR Berendes HW Isotalo L. Delayed childbearing and risk of adverse perinatal outcome. A population-based study. J Am Med Assoc. (1992) 268:886–90. 10.1001/jama.268.7.886 1640617

[18] Koshida S Arima H Fujii T Ito Y Murakami T Takahashi K. Impact of advanced maternal age on adverse infant outcomes: a Japanese population-based study. Eur J Obstet Gynecol Reprod Biol. (2019) 242:178–81. 10.1016/j.ejogrb.2019.08.011 31537416

[19] Lawlor DA Mortensen L Andersen AMN. Mechanisms underlying the associations of maternal age with adverse perinatal outcomes: a sibling study of 264,695 Danish women and their firstborn offspring. Int J Epidemiol. (2011) 40:1205–14. 10.1093/ije/dyr084 21752786

[20] Cimadomo D Fabozzi G Vaiarelli A Ubaldi N Ubaldi FM Rienzi L. Impact of maternal age on oocyte and embryo competence. Front Endocrinol. (2018) 9:327. 10.3389/fendo.2018.00327 PMC6033961 30008696

[21] Lean SC Heazell AEP Dilworth MR Mills TA Jones RL. Placental dysfunction underlies increased risk of fetal growth restriction and stillbirth in advanced maternal age women. Sci Rep. (2017) 7:9677. 10.1038/s41598-017-09814-w 28852057 PMC5574918

[22] Suzui E. Impact of the use of ultrasound scanning in prenatal examinations. Kawasaki J Med Welf. (2004) 14:59–70.

[23] Itabashi K Miura F Uehara R Nakamura Y. New Japanese neonatal anthropometric charts for gestational age at birth. Pediatr Int. (2014) 56:702–8. 10.1111/ped.12331 24617834

[24] Ashorn P Ashorn U Muthiani Y Aboubaker S Askari S Bahl R Small vulnerable newborns-big potential for impact. Lancet. (2023) 401:1692–706. 10.1016/S0140-6736(23)00354-9 37167991

[25] Hermanussen M Scheffler C. Secular trends in gestational weight gain and parity on birth weight: an editorial. Acta Paediatr. (2021) 110:1094–6. 10.1111/apa.15678 33274455

[26] Shah PS. Knowledge synthesis group on determinants of LBW/PT births. Parity and low birth weight and preterm birth: a systematic review and meta-analyses. Acta Obstet Gynecol Scand. (2010) 89:862–75. 10.3109/00016349.2010.486827 20583931

[27] Thompson JMD Clark PM Robinson E Becroft DMO Pattison NS Glavish N Risk factors for small-for-gestational-age babies: the Auckland birthweight collaborative study. J Paediatr Child Health. (2001) 37:369–75. 10.1046/j.1440-1754.2001.00684.x 11532057

[28] Langhoff-Roos J Kesmodel U Jacobsson B Rasmussen S Vogel I. Spontaneous preterm delivery in primiparous women at low risk in Denmark: population based study. Br Med J. (2006) 332:937–9. 10.1136/bmj.38751.524132.2F 16497733 PMC1444877

[29] Itoh H. Pregnancy-induced hypertension (PIH) and nutritional care in Japan. Eiyogaku Zasshi. (2011) 69:3–9. 10.5264/eiyogakuzashi.69.3

