# Supplementary material for: Sustained decline in birth weight and increased rate of preterm infants born small for gestational age in Japan
Source: Front Pediatr. 2024 Sep 30;12:1480527. doi: 10.3389/fped.2024.1480527 (PMC11472726; doi:10.3389/fped.2024.1480527)
Supplement: Supplementary file 1 [file Table1.docx]

Supplementary Material

**Table S1 Maternal and neonatal characteristics by birth year period**

| **Variables** | | | **Gestational age** | **1997–2001** | | **2002–2006** | | **2007–2011** | | **2012–2016** | | **2017–2021** | | **P-value** | **Β** |
| --- | --- | --- | --- | --- | --- | --- | --- | --- | --- | --- | --- | --- | --- | --- | --- |
| **Participants** | **All births** | n (%) | 22–24w | 3,421 | 0.06% | 4,136 | 0.08% | 4,433 | 0.08% | 4,252 | 0.08% | 3,902 | 0.09% | <0.001 |  |
|  |  |  | 25–27w | 8,925 | 0.15% | 9,623 | 0.17% | 9,326 | 0.17% | 8,490 | 0.17% | 7,023 | 0.16% | 0.009 |  |
|  |  |  | 28–31w | 25,002 | 0.43% | 25,696 | 0.47% | 24,897 | 0.46% | 22,822 | 0.45% | 19,420 | 0.44% | 0.003 |  |
|  |  |  | 32–33w | 29,838 | 0.51% | 30,143 | 0.55% | 29,750 | 0.55% | 27,775 | 0.55% | 24,079 | 0.55% | <0.001 |  |
|  |  |  | 34–36w | 238,071 | 4.06% | 239,948 | 4.35% | 239,986 | 4.48% | 222,907 | 4.41% | 191,609 | 4.37% | <0.001 |  |
|  |  |  | 37–41w | 5,565,080 | 94.80% | 5,202,492 | 94.38% | 5,052,838 | 94.25% | 4,763,565 | 94.33% | 4,135,584 | 94.38% | <0.001 |  |
|  | **Boys** | n (%) | 22–24w | 1,856 | 0.1% | 2,191 | 0.1% | 2,333 | 0.1% | 2,242 | 0.1% | 2,064 | 0.1% | <0.001 |  |
|  |  |  | 25–27w | 4,694 | 0.2% | 5,126 | 0.2% | 5,074 | 0.2% | 4,550 | 0.2% | 3,706 | 0.2% | 0.015 |  |
|  |  |  | 28–31w | 13,689 | 0.5% | 13,938 | 0.5% | 13,558 | 0.5% | 12,442 | 0.5% | 10,765 | 0.5% | <0.001 |  |
|  |  |  | 32–33w | 16,973 | 0.6% | 16,902 | 0.6% | 16,583 | 0.6% | 15,733 | 0.6% | 13,500 | 0.6% | <0.001 |  |
|  |  |  | 34–36w | 135,699 | 4.5% | 134,896 | 4.8% | 135,619 | 4.9% | 126,604 | 4.9% | 108,868 | 4.8% | <0.001 |  |
|  |  |  | 37–41w | 2,842,969 | 94.3% | 2,657,123 | 93.9% | 2,579,049 | 93.7% | 2,429,436 | 93.8% | 2,106,393 | 93.8% | <0.001 |  |
|  | **Girls** | n (%) | 22–24w | 1,565 | 0.1% | 1,945 | 0.1% | 2,100 | 0.1% | 2,010 | 0.1% | 1,838 | 0.1% | <0.001 |  |
|  |  |  | 25–27w | 4,231 | 0.1% | 4,497 | 0.2% | 4,252 | 0.2% | 3,940 | 0.2% | 3,317 | 0.2% | 0.207 |  |
|  |  |  | 28–31w | 11,313 | 0.4% | 11,758 | 0.4% | 11,339 | 0.4% | 10,380 | 0.4% | 8,655 | 0.4% | 0.408 |  |
|  |  |  | 32–33w | 12,865 | 0.5% | 13,241 | 0.5% | 13,167 | 0.5% | 12,042 | 0.5% | 10,579 | 0.5% | <0.001 |  |
|  |  |  | 34–36w | 102,372 | 3.6% | 105,052 | 3.9% | 104,367 | 4.0% | 96,303 | 3.9% | 82,741 | 3.9% | <0.001 |  |
|  |  |  | 37–41w | 2,722,111 | 95.4% | 2,545,369 | 94.9% | 2,473,789 | 94.8% | 2,334,129 | 94.9% | 2,029,191 | 95.0% | <0.001 |  |
| **Maternal age** | **All births** | y, mean (sd) | 22–24w | 29.6 | (5.2) | 30.4 | (5.2) | 30.9 | (5.4) | 31.8 | (5.7) | 32.3 | (5.5) | <0.001 | 0.678 |
|  |  |  | 25–27w | 29.6 | (5.2) | 30.5 | (5.3) | 31.3 | (5.4) | 32.2 | (5.5) | 32.5 | (5.5) | <0.001 | 0.766 |
|  |  |  | 28–31w | 29.7 | (5.1) | 30.6 | (5.2) | 31.6 | (5.4) | 32.3 | (5.5) | 32.6 | (5.4) | <0.001 | 0.753 |
|  |  |  | 32–33w | 29.6 | (5.0) | 30.5 | (5.2) | 31.4 | (5.3) | 32.1 | (5.5) | 32.5 | (5.4) | <0.001 | 0.744 |
|  |  |  | 34–36w | 29.5 | (4.8) | 30.4 | (4.9) | 31.2 | (5.1) | 31.9 | (5.3) | 32.2 | (5.3) | <0.001 | 0.693 |
|  |  |  | 37–41w | 28.9 | (4.5) | 29.6 | (4.7) | 30.5 | (5.0) | 31.2 | (5.1) | 31.5 | (5.1) | <0.001 | 0.676 |
| **Primipara** | **All births** | n (%) | 22–24w | 1,370 | 40.0% | 1,717 | 41.5% | 1,912 | 43.1% | 1,960 | 46.1% | 1,817 | 46.6% | <0.001 |  |
|  |  |  | 25–27w | 3,591 | 40.2% | 4,102 | 42.6% | 4,086 | 43.8% | 3,834 | 45.2% | 3,252 | 46.3% | <0.001 |  |
|  |  |  | 28–31w | 10,390 | 41.6% | 11,044 | 43.0% | 10,629 | 42.7% | 9,929 | 43.5% | 8,336 | 42.9% | 0.001 |  |
|  |  |  | 32–33w | 12,754 | 42.7% | 12,829 | 42.6% | 12,588 | 42.3% | 11,823 | 42.6% | 10,053 | 41.8% | 0.044 |  |
|  |  |  | 34–36w | 104,842 | 44.0% | 104,107 | 43.4% | 101,805 | 42.4% | 95,034 | 42.6% | 80,779 | 42.2% | <0.001 |  |
|  |  |  | 37–41w | 2,706,420 | 48.6% | 2,528,253 | 48.6% | 2,406,785 | 47.6% | 2,245,897 | 47.1% | 1,921,571 | 46.5% | <0.001 |  |
| **Multiple births** | **All births** | n (%) | 22–24w | 629 | 18.4% | 759 | 18.4% | 741 | 16.7% | 664 | 15.6% | 593 | 15.2% | <0.001 |  |
|  |  |  | 25–27w | 1,877 | 21.0% | 2,142 | 22.3% | 1,727 | 18.5% | 1,526 | 18.0% | 1,207 | 17.2% | <0.001 |  |
|  |  |  | 28–31w | 5,687 | 22.7% | 6,472 | 25.2% | 5,587 | 22.4% | 4,880 | 21.4% | 4,379 | 22.5% | <0.001 |  |
|  |  |  | 32–33w | 6,913 | 23.2% | 7,775 | 25.8% | 7,081 | 23.8% | 6,332 | 22.8% | 5,725 | 23.8% | 0.024 |  |
|  |  |  | 34–36w | 40,492 | 17.0% | 49,811 | 20.8% | 46,332 | 19.3% | 38,756 | 17.4% | 33,816 | 17.6% | <0.001 |  |
|  |  |  | 37–41w | 57,273 | 1.0% | 56,375 | 1.1% | 45,845 | 0.9% | 46,440 | 1.0% | 44,077 | 1.1% | <0.001 |  |
| **Birth weight** | **Boys** | g, mean (sd) | 22–24w | 593 | (109) | 577 | (109) | 577 | (107) | 559 | (109) | 548 | (111) | <0.001 | −9.837 |
|  |  |  | 25–27w | 841 | (175) | 820 | (180) | 809 | (183) | 808 | (185) | 798 | (183) | <0.001 | −11.287 |
|  |  |  | 28–31w | 1,349 | (390) | 1,290 | (315) | 1,284 | (315) | 1,272 | (306) | 1,275 | (302) | <0.001 | −14.119 |
|  |  |  | 32–33w | 1,789 | (367) | 1,755 | (343) | 1,752 | (335) | 1,733 | (329) | 1,737 | (325) | <0.001 | −10.565 |
|  |  |  | 34–36w | 2,384 | (399) | 2,356 | (390) | 2,350 | (379) | 2,352 | (377) | 2,348 | (375) | <0.001 | −7.227 |
|  |  |  | 37–41w | 3,036 | (371) | 3,014 | (368) | 3,004 | (364) | 3,002 | (363) | 3,005 | (361) | <0.001 | −6.596 |
|  | **Girls** | g, mean (sd) | 22–24w | 633 | (116) | 618 | (113) | 608 | (117) | 603 | (112) | 591 | (111) | <0.001 | −10.649 |
|  |  |  | 25–27w | 903 | (186) | 880 | (190) | 871 | (198) | 856 | (200) | 860 | (195) | <0.001 | −9.847 |
|  |  |  | 28–31w | 1,413 | (365) | 1,364 | (322) | 1,357 | (316) | 1,353 | (314) | 1,351 | (312) | <0.001 | −17.272 |
|  |  |  | 32–33w | 1,883 | (357) | 1,850 | (344) | 1,842 | (334) | 1,837 | (330) | 1,838 | (326) | <0.001 | −12.928 |
|  |  |  | 34–36w | 2,480 | (395) | 2,454 | (387) | 2,451 | (380) | 2,453 | (377) | 2,445 | (372) | <0.001 | −7.988 |
|  |  |  | 37–41w | 3,127 | (380) | 3,106 | (377) | 3,097 | (373) | 3,097 | (371) | 3,101 | (370) | <0.001 | −7.866 |
| **SGA** | **Boys** | n (%) | 22–24w | 62 | 3.3% | 105 | 4.8% | 164 | 7.0% | 158 | 7.0% | 175 | 8.5% | <0.001 |  |
|  |  |  | 25–27w | 4,257 | 3.3% | 4,483 | 4.8% | 4,322 | 7.0% | 3,770 | 7.0% | 3,118 | 8.5% | <0.001 |  |
|  |  |  | 28–31w | 12,030 | 12.1% | 12,000 | 13.9% | 11,656 | 14.0% | 10,677 | 14.2% | 9,181 | 14.7% | <0.001 |  |
|  |  |  | 32–33w | 15,472 | 8.8% | 15,231 | 9.9% | 14,927 | 10.0% | 14,149 | 10.1% | 12,232 | 9.4% | <0.001 |  |
|  |  |  | 34–36w | 129,391 | 4.6% | 128,100 | 5.0% | 128,976 | 4.9% | 120,795 | 4.6% | 104,053 | 4.4% | <0.001 |  |
|  |  |  | 37–41w | 2,790,366 | 1.9% | 2,607,280 | 1.9% | 2,534,359 | 1.7% | 2,390,833 | 1.6% | 2,076,527 | 1.4% | <0.001 |  |
|  | **Girls** | n (%) | 22–24w | 58 | 3.7% | 105 | 5.4% | 102 | 4.9% | 170 | 8.5% | 164 | 8.9% | <0.001 |  |
|  |  |  | 25–27w | 435 | 10.3% | 635 | 14.1% | 704 | 16.6% | 646 | 16.4% | 611 | 18.4% | <0.001 |  |
|  |  |  | 28–31w | 1,508 | 13.3% | 1,753 | 14.9% | 1,768 | 15.6% | 1,627 | 15.7% | 1,287 | 14.9% | <0.001 |  |
|  |  |  | 32–33w | 1,319 | 10.3% | 1,444 | 10.9% | 1,435 | 10.9% | 1,399 | 11.6% | 1,173 | 11.1% | <0.001 |  |
|  |  |  | 34–36w | 5,542 | 5.4% | 6,033 | 5.7% | 5,645 | 5.4% | 5,011 | 5.2% | 4,260 | 5.1% | <0.001 |  |
|  |  |  | 37–41w | 49,927 | 1.8% | 47,283 | 1.9% | 41,989 | 1.7% | 36,779 | 1.6% | 28,399 | 1.4% | <0.001 |  |

SGA, small for gestational age. Categorical data were assessed for trends by birth year period using the Cochran–Armitage test. The percentage of primiparous births increased linearly for preterm infants born at less than 32 weeks of gestational age and decreased for those born at 32 weeks or more. The percentage of multiple births slightly decreased at all weeks of gestation. A linear increase in the proportion of preterm births (<37 weeks) was observed, with a decrease in the proportion of term births (≥37 weeks) for boys and girls. The rate of SGA increased linearly for preterm infants born at less than 32 weeks of gestation for boys and girls. Continuous data were assessed for trends by birth year period using a linear regression analysis. A linear increase in maternal age and a linear decrease in mean birth weight were observed for all weeks of gestation.

**Table S2 Secular trend in the proportion of maternal age**

| **Gestational age** | **Maternal age, y** | **1997–2001** |  | **2002–2006** |  | **2007–2011** |  | **2012–2016** |  | **2017–2021** |  | **P–value** |
| --- | --- | --- | --- | --- | --- | --- | --- | --- | --- | --- | --- | --- |
|  |  | n | % | n | % | n | % | n | % | n | % |  |
| All participants | <20 | 91,192 | 1.6% | 90,662 | 1.6% | 71,663 | 1.3% | 61,383 | 1.2% | 38,713 | 0.9% | <0.001 |
|  | 20–24 | 832,581 | 14.2% | 683,747 | 12.4% | 580,246 | 10.8% | 439,005 | 8.7% | 354,262 | 8.1% | <0.001 |
|  | 25–29 | 2,359,146 | 40.2% | 1,855,242 | 33.7% | 1,552,594 | 29.0% | 1,354,214 | 26.8% | 1,122,948 | 25.6% | <0.001 |
|  | 30–34 | 1,929,009 | 32.9% | 2,043,549 | 37.1% | 1,961,439 | 36.6% | 1,811,867 | 35.9% | 1,589,028 | 36.3% | <0.001 |
|  | 35–39 | 587,322 | 10.0% | 742,663 | 13.5% | 1,036,899 | 19.3% | 1,132,928 | 22.4% | 1,019,315 | 23.3% | <0.001 |
|  | ≥40 | 71,087 | 1.2% | 96,175 | 1.7% | 158,389 | 3.0% | 250,414 | 5.0% | 257,351 | 5.9% | <0.001 |
| 22–24w | <20 | 113 | 3.3% | 79 | 1.9% | 111 | 2.5% | 99 | 2.3% | 54 | 1.4% | <0.001 |
|  | 20–24 | 438 | 12.8% | 497 | 12.0% | 468 | 10.6% | 360 | 8.5% | 297 | 7.6% | <0.001 |
|  | 25–29 | 1,140 | 33.3% | 1,157 | 28.0% | 1,077 | 24.3% | 939 | 22.1% | 817 | 20.9% | <0.001 |
|  | 30–34 | 1,133 | 33.1% | 1,486 | 35.9% | 1,566 | 35.3% | 1,415 | 33.3% | 1,314 | 33.7% | 0.391 |
|  | 35–39 | 512 | 15.0% | 778 | 18.8% | 1,012 | 22.8% | 1,083 | 25.5% | 1,051 | 26.9% | <0.001 |
|  | ≥40 | 85 | 2.5% | 139 | 3.4% | 199 | 4.5% | 356 | 8.4% | 369 | 9.5% | <0.001 |
| 25–27w | <20 | 237 | 2.7% | 216 | 2.2% | 165 | 1.8% | 135 | 1.6% | 98 | 1.4% | <0.001 |
|  | 20–24 | 1,271 | 14.2% | 1,102 | 11.5% | 903 | 9.7% | 678 | 8.0% | 505 | 7.2% | <0.001 |
|  | 25–29 | 2,900 | 32.5% | 2,602 | 27.0% | 2,259 | 24.2% | 1,785 | 21.0% | 1,433 | 20.4% | <0.001 |
|  | 30–34 | 2,991 | 33.5% | 3,544 | 36.8% | 3,189 | 34.2% | 2,845 | 33.5% | 2,277 | 32.4% | 0.001 |
|  | 35–39 | 1,306 | 14.6% | 1,825 | 19.0% | 2,320 | 24.9% | 2,345 | 27.6% | 2,046 | 29.1% | <0.001 |
|  | ≥40 | 220 | 2.5% | 334 | 3.5% | 490 | 5.3% | 702 | 8.3% | 664 | 9.5% | <0.001 |
| 28–31w | <20 | 558 | 2.2% | 500 | 1.9% | 376 | 1.5% | 328 | 1.4% | 228 | 1.2% | <0.001 |
|  | 20–24 | 3,287 | 13.1% | 2,737 | 10.7% | 2,276 | 9.1% | 1,665 | 7.3% | 1,269 | 6.5% | <0.001 |
|  | 25–29 | 8,443 | 33.8% | 6,953 | 27.1% | 5,850 | 23.5% | 4,864 | 21.3% | 3,965 | 20.4% | <0.001 |
|  | 30–34 | 8,337 | 33.3% | 9,580 | 37.3% | 8,515 | 34.2% | 7,660 | 33.6% | 6,436 | 33.1% | <0.001 |
|  | 35–39 | 3,701 | 14.8% | 4,967 | 19.3% | 6,401 | 25.7% | 6,321 | 27.7% | 5,620 | 28.9% | <0.001 |
|  | ≥40 | 676 | 2.7% | 959 | 3.7% | 1,479 | 5.9% | 1,984 | 8.7% | 1,902 | 9.8% | <0.001 |
| 32–33w | <20 | 655 | 2.2% | 604 | 2.0% | 469 | 1.6% | 426 | 1.5% | 239 | 1.0% | <0.001 |
|  | 20–24 | 3,865 | 13.0% | 3,212 | 10.7% | 2,765 | 9.3% | 2,121 | 7.6% | 1,663 | 6.9% | <0.001 |
|  | 25–29 | 10,432 | 35.0% | 8,434 | 28.0% | 7,238 | 24.3% | 5,997 | 21.6% | 5,024 | 20.9% | <0.001 |
|  | 30–34 | 9,986 | 33.5% | 11,099 | 36.8% | 10,495 | 35.3% | 9,423 | 33.9% | 8,223 | 34.2% | 0.132 |
|  | 35–39 | 4,136 | 13.9% | 5,678 | 18.8% | 7,199 | 24.2% | 7,510 | 27.0% | 6,655 | 27.6% | <0.001 |
|  | ≥40 | 764 | 2.6% | 1,116 | 3.7% | 1,584 | 5.3% | 2,298 | 8.3% | 2,275 | 9.4% | <0.001 |
| 34–36w | <20 | 4,059 | 1.7% | 3,924 | 1.6% | 3,211 | 1.3% | 2,831 | 1.3% | 1,855 | 1.0% | <0.001 |
|  | 20–24 | 30,757 | 12.9% | 25,219 | 10.5% | 22,144 | 9.2% | 16,968 | 7.6% | 13,563 | 7.1% | <0.001 |
|  | 25–29 | 86,700 | 36.4% | 71,497 | 29.8% | 61,345 | 25.6% | 52,109 | 23.4% | 43,090 | 22.5% | <0.001 |
|  | 30–34 | 80,461 | 33.8% | 91,025 | 37.9% | 87,406 | 36.4% | 77,932 | 35.0% | 67,081 | 35.0% | 0.368 |
|  | 35–39 | 31,005 | 13.0% | 41,422 | 17.3% | 55,280 | 23.0% | 57,577 | 25.8% | 50,760 | 26.5% | <0.001 |
|  | ≥40 | 5,089 | 2.1% | 6,861 | 2.9% | 10,600 | 4.4% | 15,490 | 6.9% | 15,260 | 8.0% | <0.001 |
| 37–41w | <20 | 85,570 | 1.5% | 85,339 | 1.6% | 67,331 | 1.3% | 57,564 | 1.2% | 36,239 | 0.9% | <0.001 |
|  | 20–24 | 792,963 | 14.2% | 650,980 | 12.5% | 551,690 | 10.9% | 417,213 | 8.8% | 336,965 | 8.1% | <0.001 |
|  | 25–29 | 2,249,531 | 40.4% | 1,764,599 | 33.9% | 1,474,825 | 29.2% | 1,288,520 | 27.0% | 1,068,619 | 25.8% | <0.001 |
|  | 30–34 | 1,826,101 | 32.8% | 1,926,815 | 37.0% | 1,850,268 | 36.6% | 1,712,592 | 36.0% | 1,503,697 | 36.4% | <0.001 |
|  | 35–39 | 546,662 | 9.8% | 687,993 | 13.2% | 964,687 | 19.1% | 1,058,092 | 22.2% | 953,183 | 23.0% | <0.001 |
|  | ≥40 | 64,253 | 1.2% | 86,766 | 1.7% | 144,037 | 2.9% | 229,584 | 4.8% | 236,881 | 5.7% | <0.001 |

Categorical data were assessed for trends by birth year period using the Cochran–Armitage test. The proportion of mothers <30 years old decreased linearly throughout the entire period, while the proportion of mothers ≥35 years old increased linearly; for the 30–34-years age group, the trend was steady or rising.

**Table S3 The rate of SGA by weeks of gestation compared between boys and girls in each birth year period**

| **Gestational age** | **sex** | **1995–1999** | | | | **2000–2004** | | | | **2005–2009** | | | | **2010–2014** | | | | **2015–2021** | | | |
| --- | --- | --- | --- | --- | --- | --- | --- | --- | --- | --- | --- | --- | --- | --- | --- | --- | --- | --- | --- | --- | --- |
|  |  | **Total, n** | **SGA,n, %** | | **P** | **Total, n** | **SGA,n, %** | | **P** | **Total, n** | **SGA,n, %** | | **P** | **Total, n** | **SGA,n, %** | | **P** | **Total, n** | **SGA,n, %** | | **P** |
| **22–24w** | **Boys** | 1,856 | 62 | 3.3% | 0.577 | 2,191 | 105 | 4.8% | 0.395 | 2,333 | 164 | 7.0% | 0.002 | 2,242 | 158 | 7.0% | 0.095 | 2,064 | 175 | 8.5% | 0.649 |
|  | **Girls** | 1,565 | 58 | 3.7% |  | 1,945 | 105 | 5.4% |  | 2,100 | 102 | 4.9% |  | 2,010 | 170 | 8.5% |  | 1,838 | 164 | 8.9% |  |
| **25–27w** | **Boys** | 4,694 | 437 | 9.3% | 0.125 | 5,126 | 643 | 12.5% | 0.024 | 5,074 | 752 | 14.8% | 0.022 | 4,550 | 780 | 17.1% | 0.367 | 3,706 | 588 | 15.9% | 0.005 |
|  | **Girls** | 4,231 | 435 | 10.3% |  | 4,497 | 635 | 14.1% |  | 4,252 | 704 | 16.6% |  | 3,940 | 646 | 16.4% |  | 3,317 | 611 | 18.4% |  |
| **28–31w** | **Boys** | 13,689 | 1,659 | 12.1% | 0.004 | 13,938 | 1,938 | 13.9% | 0.022 | 13,558 | 1,902 | 14.0% | 0.001 | 12,442 | 1,765 | 14.2% | 0.002 | 10,765 | 1,584 | 14.7% | 0.761 |
|  | **Girls** | 11,313 | 1,508 | 13.3% |  | 11,758 | 1,753 | 14.9% |  | 11,339 | 1,768 | 15.6% |  | 10,380 | 1,627 | 15.7% |  | 8,655 | 1,287 | 14.9% |  |
| **32–33w** | **Boys** | 16,973 | 1,501 | 8.8% | <0.001 | 16,902 | 1,671 | 9.9% | 0.004 | 16,583 | 1,656 | 10.0% | 0.011 | 15,733 | 1,584 | 10.1% | <0.001 | 13,500 | 1,268 | 9.4% | <0.001 |
|  | **Girls** | 12,865 | 1,319 | 10.3% |  | 13,241 | 1,444 | 10.9% |  | 13,167 | 1,435 | 10.9% |  | 12,042 | 1,399 | 11.6% |  | 10,579 | 1,173 | 11.1% |  |
| **34–36w** | **Boys** | 135,699 | 6,308 | 4.6% | <0.001 | 134,896 | 6,796 | 5.0% | 0.000 | 135,619 | 6,643 | 4.9% | <0.001 | 126,604 | 5,809 | 4.6% | <0.001 | 108,868 | 4,815 | 4.4% | <0.001 |
|  | **Girls** | 102,372 | 5,542 | 5.4% |  | 105,052 | 6,033 | 5.7% |  | 104,367 | 5,645 | 5.4% |  | 96,303 | 5,011 | 5.2% |  | 82,741 | 4,260 | 5.1% |  |
| **37–41w** | **Boys** | 2,842,969 | 52,603 | 1.9% | 0.157 | 2,657,123 | 49,843 | 1.9% | 0.125 | 2,579,049 | 44,690 | 1.7% | 0.002 | 2,429,436 | 38,603 | 1.6% | 0.247 | 2,106,393 | 29,866 | 1.4% | 0.114 |
|  | **Girls** | 2,722,111 | 49,927 | 1.8% |  | 2,545,369 | 47,283 | 1.9% |  | 2,473,789 | 41,989 | 1.7% |  | 2,334,129 | 36,779 | 1.6% |  | 2,029,191 | 28,399 | 1.4% |  |

SGA, small for gestational age, defined as birth weight <−2SD.The P value indicates the comparison of the rate of SGA between boys and girls by weeks of gestation using the chi-square test. As a whole, the rate of SGA tended to be higher in girls than in boys except at 22–24 weeks of gestation. At 28 weeks of gestation and beyond, the rate of SGA was statistically significantly higher in girls than in boys.
